# Functional Analysis of Mutations in Exon 9 of *NF1* Reveals the Presence of Several Elements Regulating Splicing

**DOI:** 10.1371/journal.pone.0141735

**Published:** 2015-10-28

**Authors:** Elisabete Hernández-Imaz, Yolanda Martín, Laura de Conti, German Melean, Ana Valero, Marco Baralle, Concepción Hernández-Chico

**Affiliations:** 1 Servicio de Genética, Hospital Universitario Ramón y Cajal, IRYCIS, Madrid, Spain; 2 Centro de Investigación Biomédica en Red de Enfermedades Raras (CIBERER), Madrid, Spain; 3 International Centre for Genetic Engineering and Biotechnology (ICGEB), Trieste, Italy; CNRS UMR7275, FRANCE

## Abstract

Neurofibromatosis type 1 (NF1) is one of the most common human hereditary disorders, predisposing individuals to the development of benign and malignant tumors in the nervous system, as well as other clinical manifestations. NF1 is caused by heterozygous mutations in the *NF1* gene and around 25% of the pathogenic changes affect pre-mRNA splicing. Since the molecular mechanisms affected by these mutations are poorly understood, we have analyzed the splicing mutations identified in exon 9 of *NF1*, which is particularly prone to such changes, to better define the possible splicing regulatory elements. Using a minigene approach, we studied the effect of five splicing mutations in this exon described in patients. These highlighted three regulatory motifs within the exon. An *in vivo* splicing analysis of an extensive collection of changes generated in the minigene demonstrated that the CG motif at c.910-911 is critical for the recognition of exon 9. We also found that the GC motif at c.945-946 is involved in exon recognition through SRSF2 and that this motif is part of a Composite Exon Splicing Regulatory Element made up of physically overlapping enhancer and silencer elements. Finally, through an *in vivo* splicing analysis and *in vitro* binding assays, we demonstrated that the c.1007G>A mutation creates an Exonic Splicing Silencer element that binds the hnRNPA1 protein. The complexity of the splicing regulatory elements present in exon 9 is most likely responsible for the fact that mutations in this region represent 25% of all exonic changes that affect splicing in the *NF1* gene.

## Introduction

Pre-mRNA splicing is a fundamental event in gene expression and thus, the synthesis of alternative-spliced isoforms of mRNA transcripts is a tightly controlled process. In addition to the conserved 5’ and 3’ splice sites at exon/intron boundaries, splice site selection also depends on different auxiliary *cis*-acting splicing regulatory elements (SREs) that recruit *trans*-acting factors and that are located in exons or introns. In classical studies, these SREs are divided into ESE and ISE (exonic/intronic splicing enhancer) elements, which favor the recognition of the nearby splice sites, and the inhibitory ESS and ISS (exonic/intronic splicing silencer) [1, 2]. Many specific RNA binding proteins (RBPs) have been identified that bind SREs in a context dependent manner. ESEs are mainly recognized by members of the SR (serine/arginine-rich) protein family [3], whereas heterogeneous nuclear ribonucleoproteins (hnRNPs) typically bind to ESSs [4]. Moreover, data from different genome-wide analyses has allowed RNA splicing maps of single RBPs to be created [5–9].

Neurofibromatosis type 1 (NF1; MIM 162200) is an autosomal dominant neurocutaneous disorder that affects 1/3,500 live births, predisposing individuals to the development of cutaneous and plexiform neurofibromas, optic nerve gliomas and other tumors of the nervous system. NF1 is caused by mutations in the *NF1* gene (*NF1*; NM_000267.2) located on chromosome 17q11.2, which spans about 350 Kb of genomic DNA. The full-length *NF1* mRNA transcript contains sixty exons, including three alternatively spliced 9br, 23a and 48a that do not involve any alteration of the gene's reading frame. Alternative splicing of exons in the *NF1* gene is highly regulated, both during development and in specific tissues. The gene encodes a major protein product of 2,818 amino acids, termed neurofibromin, which acts as a negative regulator of Ras-mediated signaling [10–12]. Indeed, *NF1* is a classic tumor suppressor gene as its biallelic inactivation causes tumor development.

According to the Human Genome Mutation Database (HGMD), more than 1,200 pathogenic mutations have been described in the *NF1* gene, including point mutations (85% to 90%), single or multiexon deletions or duplications (2%) and microdeletions encompassing *NF1* and its neighboring genes (5% to 10%). Remarkably, around 25% of pathogenic mutations in *NF1* appear to affect the correct splicing of both the constitutive and alternative exons of the gene [13–16]. In our cohort of 60 splicing mutations, 78% of them affect the conserved 5’ss (5’ splice sites) and 3’ss, while 17% occur within the coding region and the remaining 5% are located in deep intronic sequences. Currently, about 20 different changes producing exon skipping have been described in the coding sequence of *NF1* gene. However, the functional significance of many of these mutations has not been studied and the underlying molecular mechanisms are still poorly understood. In fact, only one ESE element has been characterized molecularly in exon 45 (previously named exon 37 in the historical numbering used by the NF1 consortium) where it was demonstrated that the pathogenic c.6972C>G mutation disrupts this element, impeding the binding of the YB-1 protein. In addition, this change creates an ESS that is bound by the negative splicing factors hnRNPA1, hnRNPA2 and DAZAP [17, 18].

Exon 9 of *NF1* (previously named exon 7) is particularly prone to mutations that affect splicing. Indeed, five pathological mutations (c.910C>T, c.943C>T c.945_946delinsAA, c.1007G>A and c.1039C>T) have been observed to result in aberrant splicing *in vivo* and in 4 cases confirmed *in vitro* [19–21]. Previous studies aimed to asses the splicing enhancement activity of the sequences surrounding these mutations, using several reporter minigenes. These assays showed a residual enhancement of splicing mediated by sequences encompassing the nucleotide c.910C, but the specific nature of the regulatory elements around nucleotides c.943C and c.1007G could not be assessed [20]. In this study, we have carried out an extensive mutational analysis in the regions surrounding the 5 mutations described in patients in order to elucidate the molecular mechanism affected, resulting in a better understanding of their nature as either ESE or ESS elements.

## Materials and Methods

### Minigene construction and site-directed mutagenesis

Minigene constructs were generated as described previously [22]. Briefly, exon 9 of *NF1* and the flanking intronic sequences were amplified by PCR using the primers E7D (5’-cacacactcgagAACAGCTTGTTTGGGAAGGA-3’) and E7R (5’-cacacaggatccGGCCCTAATTGCCACATTATT-3’) that each contain a 5’-XhoI and 5’-BamHI restriction site, respectively. The PCR product (680 bp) was then cloned into the XhoI/BamHI site of the pSPL3 vector to obtain the E9-pSPL3 minigene, primers were designed to introduce the mutations of interest using the QuickChange Primer Design program (the sequence of primers is available upon request). The sequence changes were introduced in the wild type minigene using the QuickChange Site-Directed Mutagenesis Kit (Agilent Technologies) according to the manufacturer’s instructions, and the fidelity of the minigenes was confirmed by sequencing.

### *In vivo* splicing assays

Different quantities of the minigenes (250–500 ng) were transfected into HeLa cells using Lipofectamine 2000 (Invitrogen), according to the manufacturer’s instructions. After 24 h, the cells were harvested and their RNA was extracted with TRIzol (Invitrogen). The total RNA (1 μg) was treated with DNAse I (Invitrogen) and reverse transcribed using the First Strand cDNA Synthesis Kit for RT-PCR (AMV; Roche). The amount of transcripts with or without exon 9 was assessed by semiquantitative PCR amplification of the cDNAs using the SD (5’ labeled with 6-FAM: 5’-TCTGAGTCACCTGGACAACC-3’) and SA (5’-ATCTCAGTGGTATTTGTGAGC-3’) primers. The RT-PCR products were separated by capillary electrophoresis on an ABI3100 (Applied Biosystems) and quantified by measuring the peak areas. Transcripts including (FL) and excluding (SK) exon 9 were assessed and the percentage of exon 9 inclusion was calculated with the formula: 100xFL/(FL+SK). All the experiments were carried out at least three times and the data were analyzed using a paired t-test.

### Over-expression of SR proteins

These analyses were performed with constructs containing the sequences of several SR proteins cloned into the pCG vector: SRSF1 (SF2), SRSF2 (SC35), SRSF3 (SRp20), SRSF4 (SRp75), SRSF5 (SRp40) or SRSF6 (SRp55). HeLa cells were co-transfected with 250 ng of the wild type or mutant E9-pSPL3 minigenes and with variable amounts of the SR-pCG constructs (0.25–1 μg) or empty pGCvector (control). The proportion of transcripts with or without exon 9 was assessed as indicated above and the data were analyzed using a t-test.

### siRNA-mediated knockdown of SRSF2 and hnRNPA1

Transfections of siRNAs into HeLa cells were performed using the Oligofectamine Reagent (Invitrogen) according to the manufacturers’ instructions. Briefly, 3 μl of siRNA (40 μM) was used in two rounds of transfection performed on two consecutive days. On the third day, wild type or mutant minigenes (500 ng) were then transfected as described above and 24 h later, the cells were harvested and divided into two aliquots to prepare RNA and protein extracts. Proteins were extracted by sonicating the lysate for 5 min at high intensity in lysis buffer: 15 mM Hepes [ph 7.5], 250 mM NaCl, 0.5% (v/v) NP-40, 10% glycerol and protease inhibitors (Roche Diagnostic). The proportion of mRNA transcripts with or without exon 9 was assessed by semi-quantative PCR and calculated as described above. A siRNA against luciferase was used as a negative control. The siRNA sequences used were the following: hnRNPA1-sense, 5’-CAGCUGAGGAAGCUCUUCAdTdT-3’, hnRNPA1-antisense: 5’-UGAAGAGCUUCCUCAGCUGdTdT-3’, SRSF2-sense: 5’- AAUCCAGGUCGCGAUCGAAdTdT-3’ and SRSF2-antisense, 5’-UUCGAUCGCGACCUGGAUUdTdT-3’. To show the effective knockdown of hnRNPA1 a Western blot was performed where tubulin was used as a loading control. SRSF2 knockdown was quantified by qPCR (see below).

### Quantitative real-time PCR

qPCR was performed to quantify the expression of SRSF2 in siRNA-mediated knockdown experiments. RNA extraction and cDNA synthesis was performed as indicated above. As control, cDNA synthesis of each sample was also performed in the absence of reverse transcriptase in order to detect genomic DNA contamination. *SRSF2* expression levels were quantified using the SYBR green technology in a CFX96 Real-Time PCR Detection System (Bio-Rad). Specific primers for *SRSF2* and *GAPDH* were designed using Beacon designer software (Bio-Rad) and the sequences are the following: SRSF2_qPCR_F: 5’-GCCGCAGCCGATCC-3’; SRSF2_qPCR_R: 5’-ACGAGGACTTGGACTTGG-3’; GAPDH_F: 5’-AAGGTGAAGGTCGGAGTCAA-3’ and GAPDH_R: 5’-AATGAAGGGGTCATTGATGG-3’. *GAPDH* was used to normalize the results. The relative expression levels were calculated using the equation ΔC_T_ = C_T(target)_−C_T(normalizer)_ for Ct normalization and the difference between ΔC_T_ test (anti-SRSF2 siRNA-treated cells) and ΔC_T_ calibrator (anti-luciferase siRNA-treated cells) were used to calculate the expression ratio and compare the expression levels [23]. The data shown are the mean fold induction ± SD from three independent experiments.

### Pull-down and Western blotting

Complementary DNA primers were designed to synthesize 20–28 bp RNA probes that spanned all the regions of interest where the mutations under study are located. The forward primer carried a T7 polymerase target sequence (5’-TACGTAATACGACTCACTATAGG-3’), while the reverse primer contained a (TG)_7_ sequence known to be a strong binding motif for TDP-43 protein (which was used for normalization in the subsequent Western blot experiments). The DNA primers were PCR amplified, purified and used as templates for T7 polymerase (Stratagene) driven transcription. The resulting RNA was treated with DNAse I (Invitrogen) and purified by phenol-chloroform. The procedures for the affinity purification of RNA-protein complexes and subsequent Western blot analysis have been described elsewhere [24].

### Electrophoretic mobility shift assay (EMSA)

Four RNA probes were synthesized according to the same procedure described above and spanning the region where the c.1007C>T mutation is located: one wild type probe (P1007G) and probes containing the three possible changes (P1007A, P1007T and P1007C). In this case 2μl of (P^32^)-UTP was included in the *in vitro* transcription reaction and the probes were purified using a Nick Column (GE Healthcare) following the manufacturer’s instructions. The protocol used for EMSA has been described in full elsewhere [24].

## Results

### Functional analysis of mutations affecting exon 9 splicing

**Fig 1:**
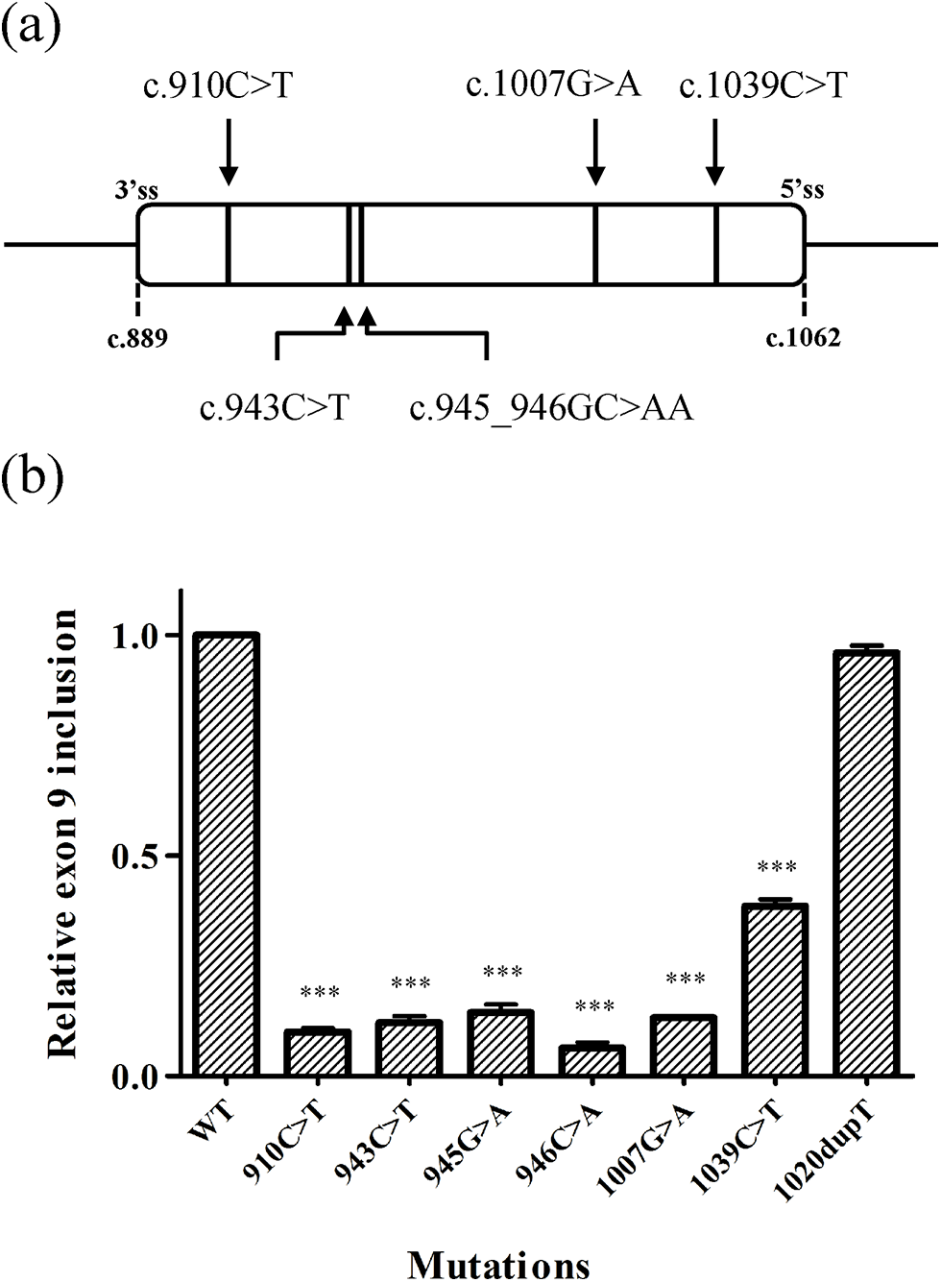
*In vivo* splicing of exon 9 in the wild type and mutant minigenes. (a) Schematic representation of the position of the mutations in *NF1* exon 9 identified in NF1 patients. (b) Comparison of the transcripts including exon 9 derived from pSPL3-minigenes. Minigenes were transfected into HeLa cells and the total RNA was purified after 24 hours. Transcripts including (FL) and excluding (SK) exon 9 were quantified by semiquantitative RT-PCR and the percentage was calculated with the formula: 100xFL/(FL+SK). All the experiments were performed at least three times (n = 3, ± SD), and the mean value of the percentage for each mutant was normalized to the wild type (WT) minigene (Y-axis). All the mutant minigenes presented a significant reduction in exon 9 inclusion (*** p<0.001), except for the c.1020dupT minigene (p = 0.14).

To date, five point mutations have been identified in the coding sequence of exon 9 of the *NF1* gene that cause exon skipping (Fig 1A): c.910 C>T (nonsense mutation) [25], c.943C>T (nonsense mutation) [26], the double mutation c.945_946delinsAA (synonymous and missense changes, respectively) [19], c.1007G>A (nonsense mutation) [26], and c.1039C>T (nonsense mutation) [21]. Given these mutations are not located in close proximity; it is likely that each affect different SREs involved in exon 9 recognition. We therefore performed a functional analysis of the mutations in a minigene context in order to characterize the potential SREs in this exon.

To generate the E9-pSPL3 minigene, the coding sequence of exon 9 spanning from c.889 to c.1062 and the flanking intronic sequences (258 bp upstream of the exon and 248 downstream of the exon) were cloned into the pSPL3 vector as described previously [22]. All the mutations studied were introduced into the minigene by site-directed mutagenesis, and for the c.945_946delinsAA mutation two different constructs were generated in order to analyze the effect of each change independently (c.945G>A and c.946C>A). Each of the six minigenes was transfected into HeLa cells and expressed for 24 h. The total mRNA was then extracted from the cells and the effect of each mutation was analyzed by RT-PCR, calculating the proportion of transcripts with or without exon 9 in each case. Exon 9 of *NF1* was included in the majority of the transcripts derived from the wild type minigene, ranging from 65% to 81%. By contrast, the inclusion of exon 9 decreased significantly for all the mutant minigenes (Fig 1B). A control minigene was also tested that harbors the c.1020dupT mutation that does not affect exon 9 splicing *in vivo* and the proportion of transcripts containing exon 9 was similar to those of the wild type minigene.

### Characterization of SREs in exon 9

**Fig 2:**
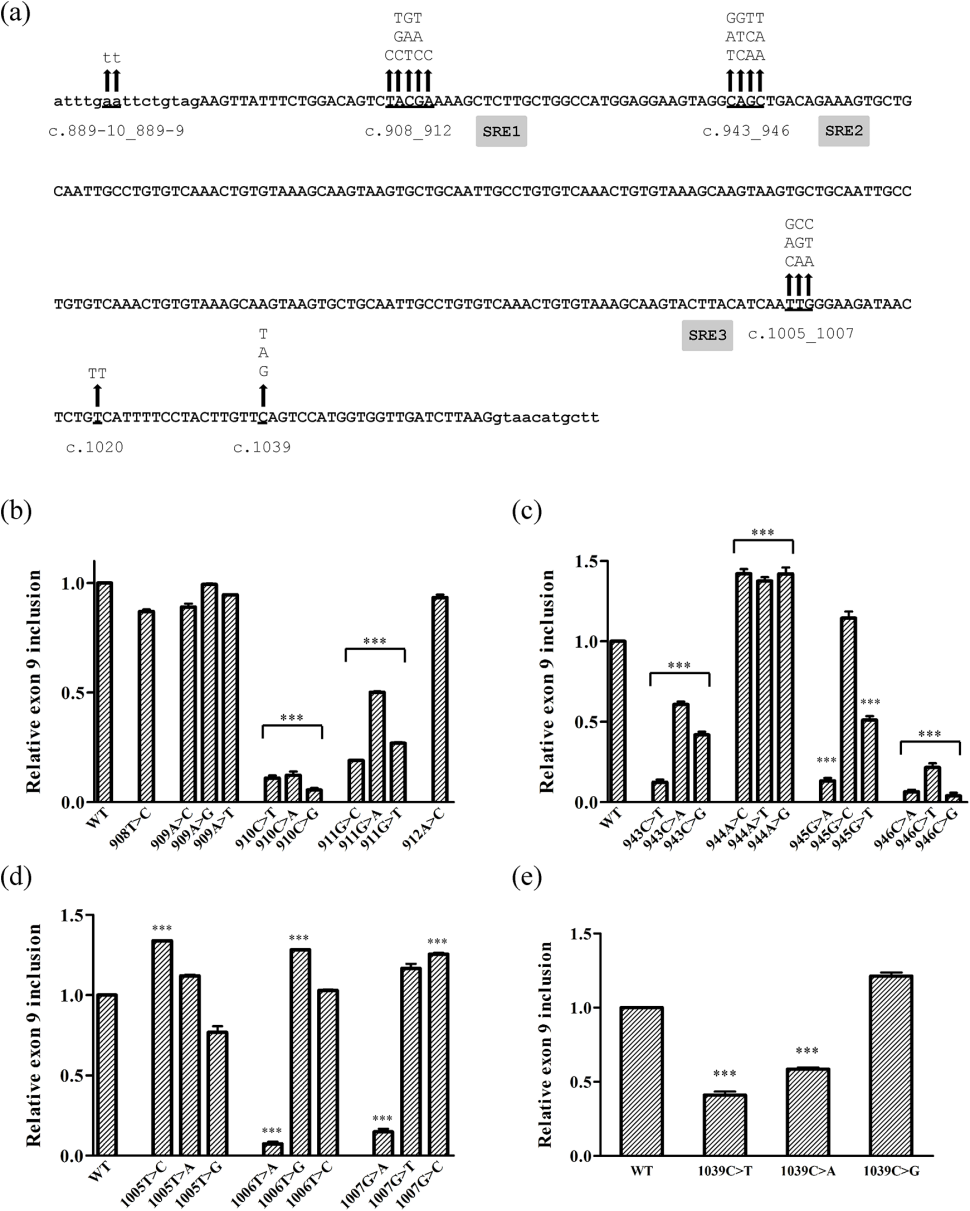
Functional analysis of SREs in *NF1* exon 9. (a) Summary of the mutations generated in the E9_pSPL3 minigene. Intronic and exonic sequences are shown in lowercase and uppercase, respectively. The arrows indicate the nucleotide positions substituted and the changes generated. The three putative SREs are shown in grey. Four studies were performed (SRE1, SRE2, SRE3 and the position c.1039) and, for each study, the proportion of transcripts including exon 9 from each mutant minigene was assessed and normalized to the wild type minigene as previously described (Fig 1B). Mutant minigenes that presented a significant reduction in exon 9 inclusion are indicated (*** p<0.001). (b) Study of SRE1. Changes were introduced into the c.908_912 region. (c) Study of SRE2. Changes were introduced into the 943_946 region. (d) Study of SRE3. Changes were introduced into the c.1005_1007 region. (e) Study of position c.1039. Three different changes were tested at c.1039.

Having demonstrated that the mutations provoked aberrant splicing of exon 9 in the minigene context, we set out to better define the SREs that were being affected, generating a total of 40 changes in the E9-pSPL3 minigene (Fig 2A). Firstly, all three possible substitutions were introduced at position c.910, as well as mutating the nucleotides from c.908 to c.912 (Fig 2B). There was a significant reduction in the proportion of transcripts including exon 9 produced from the minigenes carrying any change at position c.910, and a similar effect was observed when the changes were introduced at position c.911. By contrast, no effect was observed for the substitutions at c.908, c.909 and c.912. These observations indicate that any change at the c.910_911 CG dinucleotide disrupts the putative ESE motif (SRE1).

The SRE element accentuated by the c.943C>T and c.[945G>A;946C>A] mutations was characterized in an analogous manner to SRE1, testing a total of 12 changes. All the changes introduced at position c.943, c.945 (except c.945G>C) and c.946 significantly reduced exon 9 inclusion, whereas all three changes at c.944 significantly enhanced its inclusion (Fig 2C). Accordingly, the c.943_946 region appears to contain a composite exonic splicing regulatory element (CERES), where there is a physical overlap of an ESE and ESS elements (SRE2) as it is unlikely that the 3 nucleotide substitutions at position c.944 would all result in the creation of an ESE.

A total of nine changes were tested around the pathogenic c.1007G>A mutation, and only the c.1006T>A change and the pathogenic mutation itself significantly hindered the inclusion of exon 9 (Fig 2D). The c.1007G>A mutation creates a UAGGAA motif (c.1006_1011), which shows strong homology to the hnRNPA1 protein binding motif (UAGGGA), a protein that typically binds to ESSs and that inhibits exon recognition. Indeed, the c.1006T>A mutation generates an identical sequence (c.1005_1010) to the hnRNPA1 binding site. These results suggest that an ESS element is created by the c.1007G>A mutation that would recruit the hnRNPA1 protein and provoke the exon 9 skipping observed (SRE3).

Finally, all three possible substitutions were generated at the position of the c.1039C>T mutation (Fig 2E). This pathogenic mutation and the c.1039C>A change moderately diminished the inclusion of exon 9, while the c.1039C>G change produced a significant increase in the transcripts carrying this exon. As such, it was not clear in this case whether the c.1039C>T mutation affected an ESE or if it created an ESS.

A list of the mutations analyzed and a summary of their effects on splicing is shown in S1 Table.

### SREs in exon 9 are necessary due to a weak 3’ splice site

**Fig 3:**
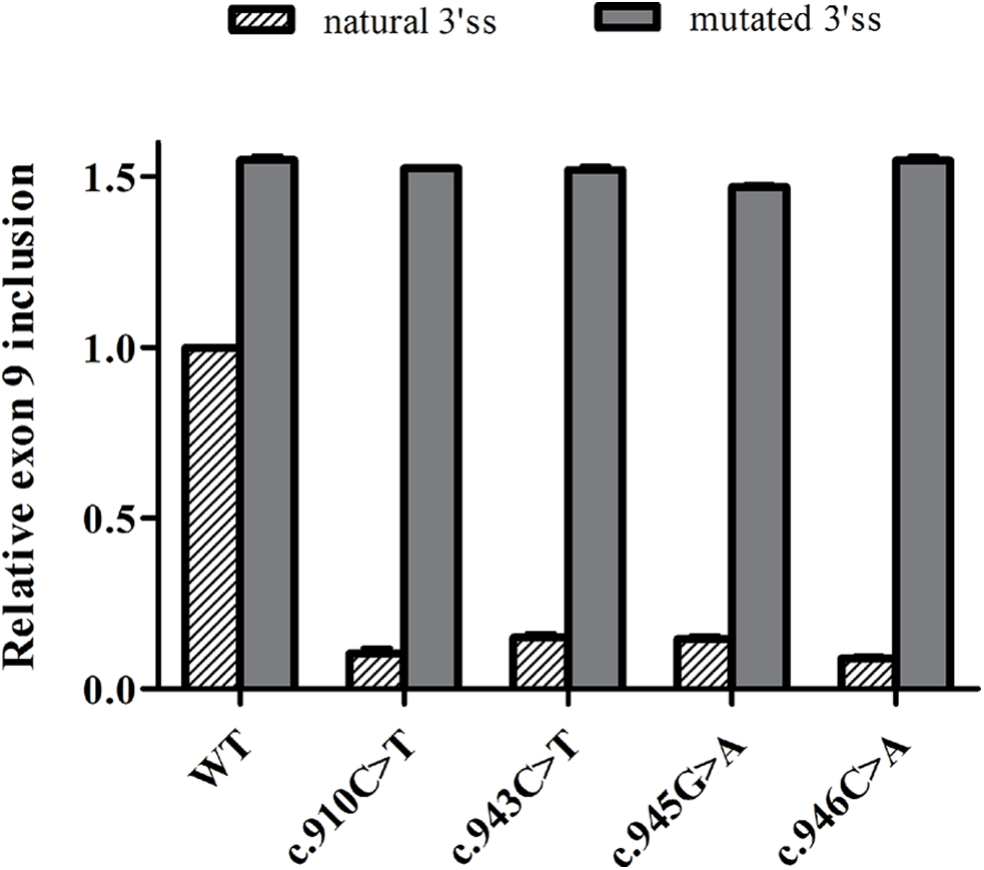
Effect of the strength of the acceptor splice site in exon 9 splicing. The natural 3’ss of the exon 9 in each minigene was substituted with a more functional one by site-directed mutagenesis (c.889-10_889-9delinsTT). The proportion of transcripts including exon 9 was assessed and normalized to the wild type minigene. The stripped and solid bars represent the exon 9 inclusion from the different minigenes with the natural 3’ss and the 3’ss mutated, respectively. All the differences were statistically significant (*** p<0.001).

It is known that ESE elements are commonly present in exons with weak splice sites [27], yet an analysis of the strength of the 5’ss using different *in silico* programs indicated that the donor splice site of exon 9 is optimal (SSPNN: 0.93; MAXENT: 8.82; MDD: 12.68; MM: 6.99; WMM: 7.53). By contrast, the 3’ss revealed a weak exon 9 acceptor site, probably due to its suboptimal polypirimidine tract (SSPNN: 0.64, MAXENT: 3.66, MM: 3.74, WMM: 4.5). Therefore, the natural 3’ss of this exon was substituted with a more functional one by introducing the c.889-10_889-9delinsTT mutation into the minigene, thereby increasing the SSPNN score from 0.64 to 0.98. As expected, this modification increased the proportion of transcripts carrying exon 9 and it was included in virtually all transcripts (increasing from 68% to > 99%: S1 Table). Furthermore, all four pathogenic mutations tested in the modified 3’ss minigene no longer inhibited the inclusion of exon 9, which in fact increased more than 10-fold (Fig 3), suggesting the presence of ESE elements that favor exon 9 recognition.

### Analysis of SR proteins controlling exon 9 inclusion

**Fig 4:**
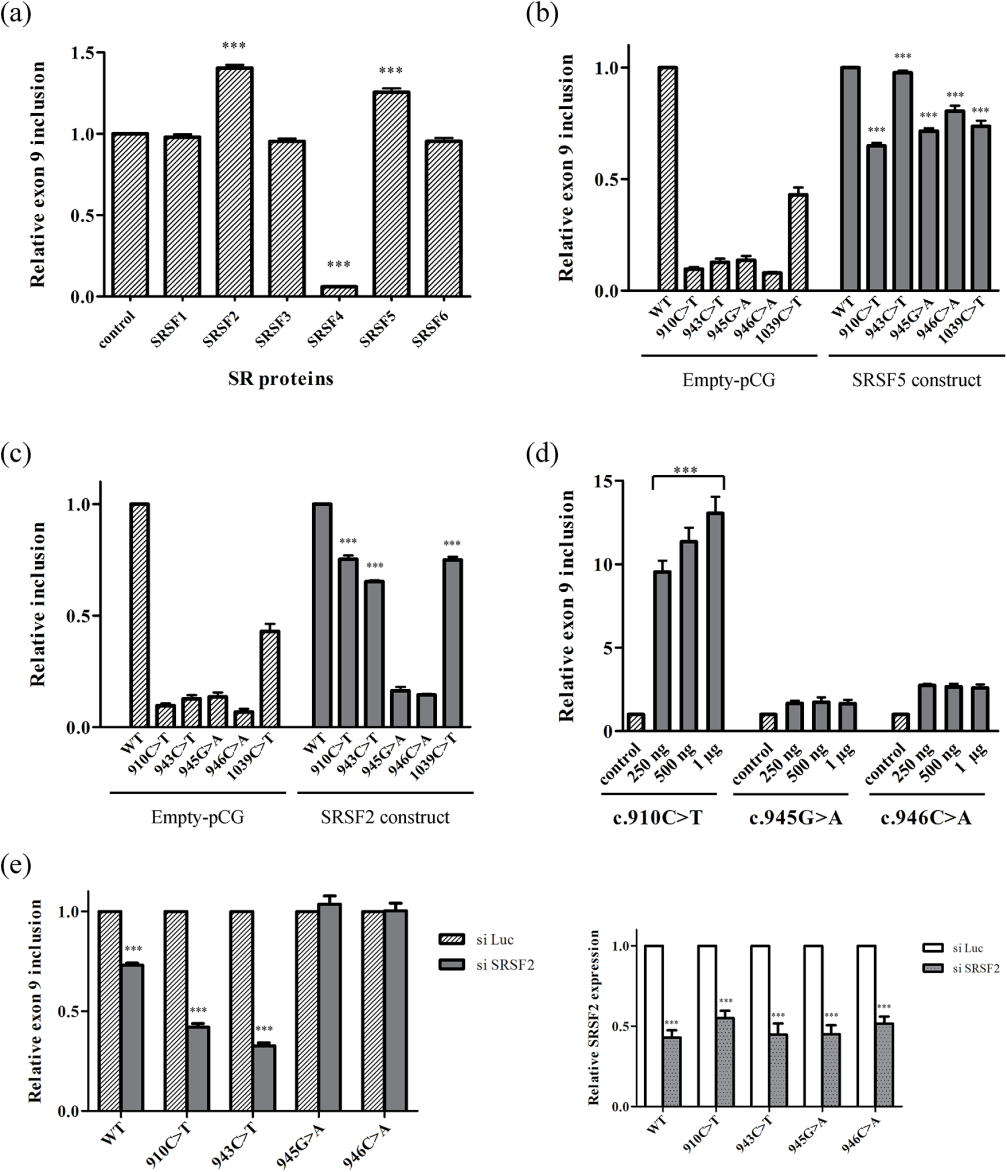
Analysis of SR proteins that regulate the inclusion of exon 9. Different SR expression constructs cloned in the pGC vector were co-transfected with the pSPL3-minigenes in HeLa cells to test their effect on *NF1* exon 9 splicing. (a) Effect of the overexpression of SR proteins on the wild type minigene. The proportion of transcripts including exon 9 was normalized to the assay of HeLa cells co-transfected with the wild-type pSPL3-minigene and the empty pGC vector (control). The overexpression of SRSF2 and SRSF5 enhanced significantly the inclusion of exon 9. (b) Effect of the overexpression of SRSF5 (SRp40) protein on the mutant minigenes. Comparison of the proportion of transcripts containing exon 9 cotransfecting the minigenes with the empty pCG vector (stripped bars) and with the SRSF5 construct (grey bars). The values have been normalized to the wild type assays. The overexpression of SRSF5 enhanced the inclusion of exon 9 in all the mutant minigenes. (c) Effect of the overexpression of SRSF2 (SC35) protein on the mutant minigenes. Comparison of the proportion of transcripts containing exon 9 in experiments co-transfecting the minigenes with the empty pCG vector (stripped bars) or with SRSF2 constructs (grey bars). The values have been normalized to the wild type assays. Just c.945G>A and c.946C>A mutant minigenes failed to augment the inclusion of exon 9 when SRFS2 was overexpresed. (d) Effect of the increase of SRSF2. pSPL3 mutant minigenes were co-transfected with empty pGC (control, stripped bars) and with increasing amounts of SRSF2 constructs (grey bars). The proportions of transcripts containing exon 9 have been normalized to the controls. SRSF2 rising has no effect on the inclusion of exon 9 from c.945G>A and c.946C>A mutant minigenes whereas there is a gradual increase in the inclusion from c.910C>T mutant. (e) Relative quantification of transcripts including exon 9 upon SRSF2 knockdown. The proportions of transcripts from the minigenes are indicated relative to their respective luciferase controls (stripped bars). Transcripts derived from the WT, c.910C>T and c.943C>T minigenes have a significant reduction in exon 9 inclusion (*** p>0.001) while no effect is observed for c.945G>A and c.946C>A mutant minigenes. In the right panel the qPCR results for the SRSF2 expression levels are shown. SRSF2 levels upon SRSF2 siRNA treatment (grey dotted bars) have been normalized to the SRSF2 levels upon luciferase siRNA treatment (white bars) confirming the effective silencing (*** p<0.001).

To identify the SR proteins that could control exon 9 inclusion through binding to the SREs identified, we tested the effect of six SR proteins on exon 9 splicing using wild type and mutant minigenes. We initially assessed *in vivo* splicing in HeLa cells transfected with the E9-pSPL3 minigene and mammalian expression constructs encoding SRSF1 (SF2), SRSF2 (SC35), SRSF3 (SRp20), SRSF4 (SRp75), SRSF5 (SRp40) or SRSF6 (SRp55). In the presence of the SRSF2 and SRSF5 proteins there was a strong increase in the proportion of transcripts containing exon 9 (Fig 4A), indicating that these two SR proteins could assemble on ESE sequences to promote splicing. By contrast, SRSF4 expression strongly diminished the inclusion of exon 9, indicating it had a silencing effect on exon 9 recognition. Indeed, it is known that some SR proteins can inhibit exon recognition depending on the context and the sequence to which they bind [28, 29].

Disrupting the putative SRSF5 and SRSF2 binding sites would abrogate the stimulatory effect of the corresponding SR protein. Through *in vivo* splicing assays with the mutant minigenes, we attempted to identify the ESE sequences that interact with SRSF5 and SRSF2. Co-transfection with SRSF5 enhanced the inclusion of exon 9 in all the mutant minigenes tested (Fig 4B), indicating that inclusion was not mediated by any of the sequences in which the pathological mutations were present. However, SRSF2 could not augment the presence of exon 9 in the c.945G>A and c.946C>A mutant minigenes (Fig 4C), while it increased its retention in the other three mutant minigenes tested carrying the pathological mutations, including c.943C>T. Furthermore, co-transfection of increasing amounts of the construct encoding SRSF2 with c.910C>T mutant minigene (from 250 ng to 1 μg) gradually enhanced the inclusion of exon 9, whereas no such effect was observed with the c.945G>A and c.946C>A mutant minigenes (Fig 4D).

To further confirm the implication of SRSF2 in exon 9 recognition, this protein was silenced in HeLa cells and the splicing of exon 9 was assessed in the wild type and four mutant minigenes (Fig 4E). The inclusion of exon 9 from the wild type minigene decreased by 25% upon SRSF2 silencing, and a similar effect was observed when the c.910C>T and c.943C>T minigenes were analyzed. Interestingly, SRSF2 silencing did not produce any change in the inclusion of exon 9 in the transcripts derived from either the c.945G>A or c.946C>A minigenes, further evidencing that both these mutations disrupt the binding of SRSF2. Overall, these results indicate that the GC dinucleotide at position c.945_946 is part of a splicing enhancer motif, SRE2, to which the SRSF2 protein binds. To demonstrate the binding of SRSF2 to exon 9, pull-down assays were performed and the protein extracts obtained were probed in Western Blots with an antibody against SRSF2. For these experiments, four different RNA probes were synthesized (the wild type P943_946 and three mutant probes: P943T, P945A and P946A), yet surprisingly, no SRSF2 binding was detected even when the stringency conditions were modified to favor the formation of RNA-protein complexes (data not shown).

### The c.1007G>A mutation creates a novel ESS element recognized by hnRNPA1

**Fig 5:**
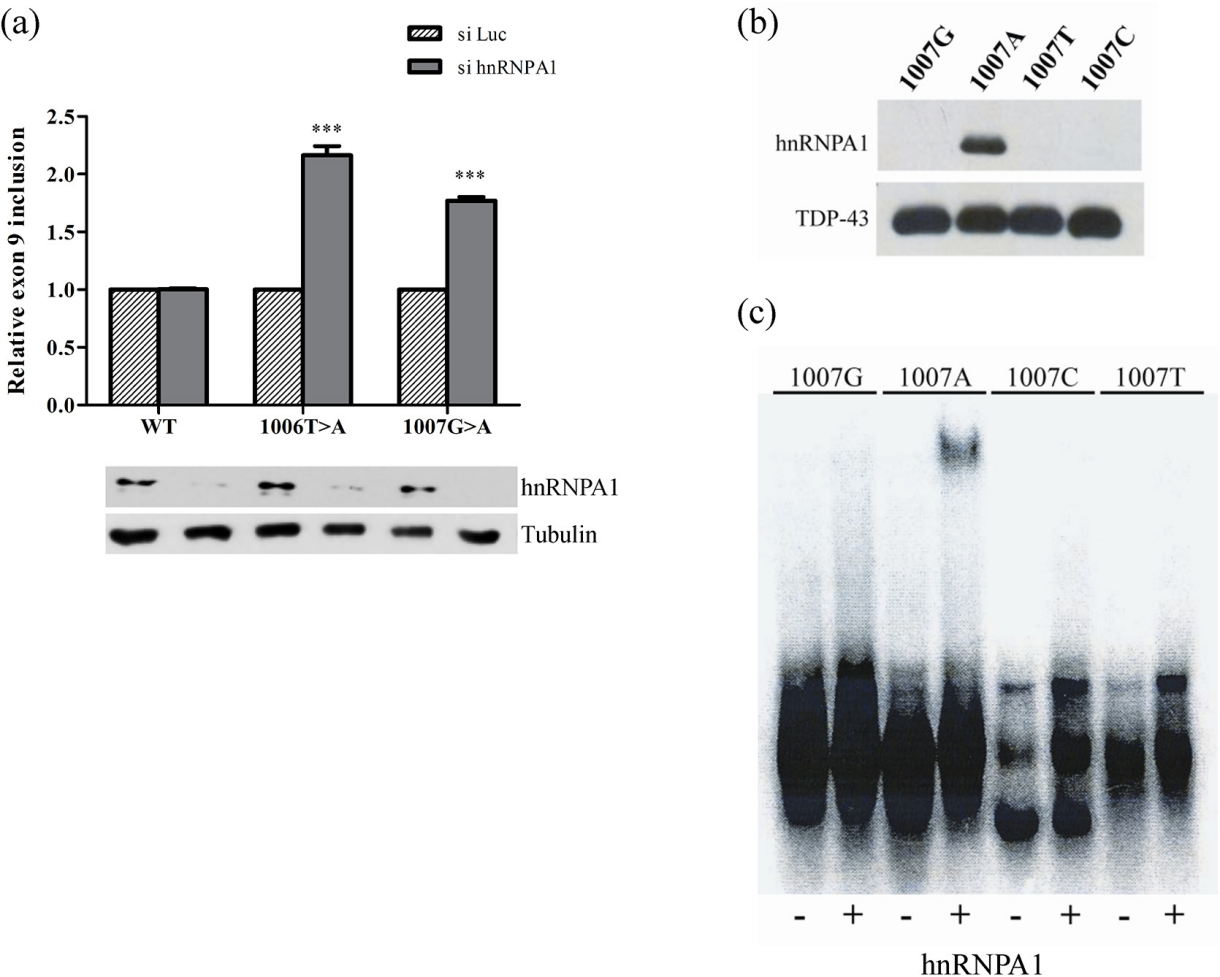
Characterization of hnRNPA1 binding to SRE3. (a) Relative quantification of transcripts including exon 9 upon hnRNPA1 knockdown. The proportion of transcripts is indicated relative to the luciferase controls (stripped bars). The lower panel shows the western blot of the knockdown of hnRNPA1. Tubulin was used as loading control. Silencing of hnRNPA1 increased the inclusion of exon 9 from the c.1006T>A and c.1007G>A mutant minigenes (*** p<0.001). (b) Western blot of the pull-down samples probed with an antibody against hnRNPA1. Four RNA probes of 24 bp spanning the c.1007 position were used to pull-down the proteins: 1007G (wild type), 1007A, 1007T and 1007C (three mutations, respectively). Only the probe containing the c.1007G>A mutations was able to bind to the hnRNPA1 protein. (c) EMSA assay using the recombinant hnRNPA1 protein and the same four probes used for the pull-downs, showing that only this protein binds to the radioactive 1007A probe.

To determine whether the c.1007G>A mutation could create an hnRNPA1-dependent ESS, we performed *in vitro* splicing assays using the minigenes to test the effect of hnRNPA1 overexpression and the effect of siRNA-mediated silencing of this protein. We found that co-transfecting HeLa cells with the E9-pSPL3 wild type and the c.1007G>A mutant minigene, along with a construct encoding hnRNPA1, produced similar rates of exon 9 inclusion in the transcripts derived from the minigenes (data not shown). The failure of hnRNPA1 overexpression to affect exon 9 inclusion was probably due to the high level of the constitutive hnRNPA1 protein in the cells. On the other hand, when the protein was silenced in HeLa cells before transfecting the minigenes no effect was observed on wild type minigene splicing. By contrast, silencing of hnRNPA1 increased the inclusion of exon 9 for the c.1006T>A and c.1007G>A mutant minigenes around 2.1-fold and 1.77-fold, respectively (Fig 5A). These results indicate the c.1007G>A pathogenic mutation creates a binding site for hnRNPA1 which in turn represses exon 9 recognition.

To demonstrate that the c.1007G>A mutation creates an ESS motif that binds the hnRNPA1 protein (SRE3), a pull-down assay and Western blotting was carried out using four different probes: the wild type P1007G probe and three mutant P1007A, P1007T and P1007C probes (Fig 5B). The hnRNPA1 protein only bound to the P1007A probe, as confirmed in an electrophoretic mobility shift assay **(**EMSA) in which the formation of a retarded own complex between the 1007A probe and the recombinant hnRNPA1 protein was observed (Fig 5C). Overall, these results confirm the c.1007G>A pathogenic mutation creates a binding site for hnRNPA1 which in turn represses exon 9 recognition.

## Discussion

It is estimated that around one third of mutations affect the correct splicing of pre-mRNAs [30], indeed around 25% of known *NF1* changes produce splicing alterations. The elevated ratio of splicing mutations reflects the need to characterize pathogenic changes at the cDNA level since splicing mutations arising outside the conserved 5’ and 3’ splice sites could easily be misclassified as coding variants if only the genomic DNA is studied. The alterations to the coding sequence of *NF1* that produce exon skipping are particularly interesting, potentially providing valuable information regarding the SREs beyond the 5’ and 3’ splice sites that regulate splicing. We performed an extensive functional analysis of the splicing mutations identified in the coding sequence of *NF1* exon 9, mainly to understand the molecular mechanisms affected by the alterations observed.

To better understand the SRE elements present in exon 9, we generated 40 different mutations in the E9-pSPL3 minigene and studied the effect of all these changes on the splicing outcome. In addition, we attempted to identify the SR and hnRNP proteins involved in exon 9 recognition. The analysis of eleven mutations created in the c.908_912 region indicated that disruption of one ESE (SRE1) is responsible for the exon skipping observed. However, we couldn’t identify the proteins that would bind to this region, even after analyzing a battery of SR and hnRNP proteins. As the spliceosome is composed of more than 150 proteins [31], we focused our study on the most frequent *trans-*acting factors.

In addition, we analyzed the c.943_946 region of SRE2 creating a total of 12 mutations. As postulated previously, the results obtained here indicated that SRE2 is involved in a complex interaction between positive and negative regulators -CERES- as some of the mutations produce exon skipping and others significantly increase the inclusion of this exon. The *in vivo* splicing assays performed with SR-encoding constructs clearly showed the critical role of the CG motif at position c.945_946 for SRSF2’s enhancing effect. Although the 5’-GCUG-3’ sequence at position c.945_948 matches the 5’-SSNG-3’ high-affinity binding sequence identified for SRSF2 [32], we failed to demonstrate SRSF2 binding *in vitro*. While this might reflect technical problems, it is also possible that SRSF2 does not bind directly to the RNA but rather, that it interacts with an unidentified protein that itself binds directly and that is an absolute requirement for the inclusion of exon 9. This mechanism has been described previously for exon 10 of the *tau* gene, where hnRNPG has been shown to bind the SRSF4 (SRp75) protein to the Intronic Splicing Silencer (ISS) element found in the downstream intron. The complex formed by both proteins is necessary for the silencing effect of the ISS [33, 34]. Moreover, hnRNPG and its paralogue RBM promote the inclusion of exon 7 of *SMN2* and these two proteins bind directly to Htra2-β1, a splicing factor that stimulates the inclusion of this exon 7 by binding to its AG-rich ESE in the pre-mRNA [35]. The complex nature of the SRE2 motif should also be borne in mind, where it is likely that several factors participate in splicing regulation in close spatial proximity to one another. Thus, constant competition between positive and negative factors will determine whether the exon is included in the transcripts or not.

At SRE3, in the c.1007 region, it has previously been shown that a 13 bp sequence spanning c.1000_1012 did not significantly augment the inclusion of exon 2 in the pSXN minigene [20]. Indeed, the c.1007G>A mutation was proposed to generate an ESS or contribute to a CERES. Elsewhere, it was shown that the c.1005T>C change enhanced the inclusion of exon 9 [22]. Here, we found that some mutations in the c.1005_1007 region increased the inclusion of exon 9 and thus, we cannot rule out the presence of a CERES element in this region. However, only the pathogenic c.1007G>A mutation and the c.1006T>A change significantly diminishes exon inclusion, creating a binding site for hnRNPA1, so it would appear that an ESS element is indeed generated. In fact, both RNA-protein binding assays (Western Blot and EMSA) and functional experiments (protein silencing) indicate that the pathogenic c.1007G>A mutation creates a binding site for hnRNPA1, which in turn dampens exon 9 recognition and provokes skipping of the exon.

Other mutations have been described in different genes with a similar effect on exon splicing. In the *SMN1/SMN2* genes three natural variants are involved in regulating exon 7 splicing. Two of these are nucleotide differences between *SMN1* and *SMN2* that contribute to the inefficient splicing of the *SMN2* exon 7: the +6 C>T transition in exon 7 disrupts a SF2/ASF-ESE [36] and/or creates an hnRNPA1-ESS [37]; and the +100A>G in intron 7 creates an hnRNPA1-ISS [37]. Another natural variant of *SMN2* that occurs infrequently in SMA patients is c.859G>C in exon 7, a variant that enhances the inclusion of the SMN2 exon 7 in milder SMA patients by creating a SF2/ASF-ESE [38] or by disrupting an hnRNPA1-ESS [39]. Furthermore, a transversion occurring at position +6 in exon 18 of the *BRCA1* gene disrupts an ESE that is bound to by the ASF/SF2 splicing factor, in turn creating an ESS that binds hnRNPA1 (and also hnRNPA2 and DAZAP), the primary determinant of exon 18 skipping [40]. In a similar manner, a nonsense mutation in exon 39 of *DMD* creates a binding site for hnRNPA1 that provokes the exclusion of this exon [41]. These, along with other examples in the literature, show the importance of hnRNPA1-mediated exon skipping events, and they illustrate the difficulties in defining the underlying mechanisms.

In summary, we have carried out an extensive analysis of splicing mutations in the sequence encoding exon 9 of *NF1*, revealing the presence of different SREs that control the expression of this exon. The description of such specific regulation of splicing will help provide a better understanding of the complex regulatory programs directing pre-mRNA processing. Moreover, it highlights the extreme complexity behind exon recognition in the *NF1* gene, which more than likely occurs in several of its exons and underlies the high rate of mutations affecting splicing in this gene.

## Supporting Information

S1 Table Summary of the mutations analyzed. (PDF)
