# Supplementary material for: Functional Analysis of Mutations in Exon 9 of NF1 Reveals the Presence of Several Elements Regulating Splicing
Source: PLoS One. 2015 Oct 28;10(10):e0141735. doi: 10.1371/journal.pone.0141735 (PMC4624989; doi:10.1371/journal.pone.0141735)
Supplement: S1 Table — (PDF) [file pone.0141735.s001.pdf]

S1 Table. Summary of the mutations analyzed.

Region	Position in exon 9	WT	Mutants					
		nt	change	% ± SD	change	% ± SD	change	% ± SD
**SRE1**	c.908	T	C	57,8 ± 0,9	-	-	-	-
	c.909	A	C	59,1 ± 1,5	G	66 ± 0,4	T	62,8 ± 0,3
	c.910	C	T	7 ± 1,5	A	8,2 ± 1,6	G	3,7 ± 0,9
	c.911	G	C	12,6 ± 0,1	A	33,3 ± 0,4	T	17,9 ± 0,4
	c.912	A	C	62 ± 1,3	-	-	-	-
**SRE2**	c.943	C	T	8,5 ± 2,1	A	41,9 ± 2,3	G	28,8 ± 1,9
	c.944	A	C	95,1 ± 2,9	T	93,8 ± 1,1	G	96,7 ± 0,3
	c.945	G	A	10,1 ± 1,3	C	77,9 ± 2,6	T	33,8 ± 2,7
	c.946	C	A	4,4 ± 1,6	T	14,7 ± 2	G	2,7 ± 1,9
**SRE3**	c.1005	T	C	95,4 ± 1,5	A	79,4 ± 0,7	G	52 ± 1,4
	c.1006	T	A	5,2 ± 1,4	G	91,3 ± 0,9	C	72,3 ± 1
	c.1007	G	A	10,5 ± 2,5	T	81,9 ± 2,3	C	88,2 ± 0,2
	c.1039	C	T	27,3 ± 2,2	A	38,9 ± 1	G	84,9 ± 2,4
	c.1020	T	TT	63,7 ± 1,6	-	-	-	-

	Minigene	3'ss nat	3'ss opt
**3'ss mutants**	WT	68,2 ± 3,5	99,15 ± 0,6
	c.910C>T	6,7 ± 1,7	97,6 ± 0,6
	c.943C>T	9,31 ± 1,9	97,3 ± 0,4
	c.945G>A	9,91 ± 1,5	94,1 ± 0,3
	c.946C>A	5,2 ± 1,2	99,2 ± 0,2

The splicing outcome of each mutant minigene is shown as "%" and indicates the mean value of the percentage of inclusion of at least 3 independent experiments.
SD: standard deviation; nt: nucleotide; 3'ss nat: natural 3'ss; 3'ss opt: optimized 3'ss.
